# Does tranexamic acid diminish hemorrhage and pain in open elbow arthrolysis? a systematic review and meta-analysis

**DOI:** 10.1186/s12891-023-06835-7

**Published:** 2023-10-06

**Authors:** Mohammad Hadi Nejat, Amirhosein Khayami, Mahla Daliri, Mohammad H. Ebrahimzadeh, Masoumeh Sadeghi, Ali Moradi

**Affiliations:** 1 Orthopedics Research Center, Ghaem hospital, Mashhad University of Medical Sciences, Mashhad, Iran; 2 Department of Epidemiology, Faculty of Health, Mashhad University of Medical Sciences, Mashhad, Iran

**Keywords:** Tranexamic acid, Elbow arthrolysis, Blood loss, Drain output

## Abstract

**Background/Objective:**

: Effective hemostasis has the potential to reduce inflammation and pain, leading to potential benefits in the early rehabilitation of patients who undergo elbow arthrolysis. In the present study, we aim to assesse the effects of tranexamic acid (TXA) on elbow arthrolysis postoperative blood loss, patients’ pain perception according to the visual analog scale (VAS), elbow range of motion (ROM), and complications.

**Methods:**

We systematically searched PubMed, Web of Science, SCOPUS, and Cochrane Library. We included controlled trials, either randomized (RCT) or non-randomized studies of intervention (NRSI) comparing the effects of intravenous tranexamic acid (TXA) treatment with placebo/no treatment on postoperative blood loss, pain VAS score, elbow ROM, and complications, in patients who underwent open or closed elbow arthrolysis surgery.

**Results:**

One RCT, and three NRSIs met eligibility criteria. The meta-analysis determined that tranexamic acid application reduced drain output 34 mm on average (WMD: -34.00; 95% CI: -49.45, -18.55). There was a discrepancy among included articles in terms of intra-operative blood loss; although the study with the largest sample size (291 and 296 patients in the case and control groups, respectively) reported reduced intra-operative blood loss in patients who received TXA. The pooled estimation for the pain VAS score on the first day post-operatively indicates a reduction in pain among patients in the TXA group (WMD: -0.82; 95% CI: -1.36, -0.28). Results for ROM, and complications’ rate such as hematoma and ulnar nerve palsy were not different between the two groups.

**Conclusion:**

TXA may be beneficial to reduce elbow arthrolysis bleeding volume. However, it dose not seem to affect final elbow ROM and patients’ pain score. Further high-quality clinical trials are needed to draw a robust conclusion on this topic.

**Supplementary Information:**

The online version contains supplementary material available at 10.1186/s12891-023-06835-7.

## Introduction

Background: Post-traumatic elbow stiffness occurs in 56% of patients after elbow trauma and affects millions of people worldwide [1–3]. One of the surgical options for treating elbow contracture is elbow arthrolysis [4]. Achieving effective hemostasis In elbow arthrolysis has the potential to alleviate inflammation and pain, thereby offering potential advantages for the early postoperative rehabilitation of patients undergoing the procedure [5, 6]. In fact, when there is bleeding, inflammatory cells migrate to the surgical site and therefore can cause pain, arthrofibrosis,heterotopic ossification or inflammation. Inflammation of synovium by itself could cause sensitivity to microtraumas and so cause bleeding, which finally causes cartilage destruction [5–7]. Therefore, sufficient hemostasis accelerates the physical therapy progression, makes the discharge of patients earlier, reduces blood transfusion, and reduces morbidity due to perioperative hemorrhage [5–7]. In addition, blood loss or hemorrhage is the most common complication of surgical deaths, which doubles the importance of effective hemostasis [8].

Tranexamic acid (TXA) is an antifibrinolytic agent that was first implied in the management of abnormal bleeding conditions, such as hyperfibrinolysis in Japan in 1962 [9, 10]. TXA is a lysine-derivate agent which competitively inhibits activation of plasminogen, therefore interrupts clot formation cascade and decreases blood loss [11]. For this reason, surgeons widely use TXA in cardiac, gastrointestinal, and gynecologic procedures [10]. Using TXA in orthopedic procedures regarding its benefits had risen from 0% to 2006 to 11.2% in 2012 [8]. The efficacy of topical and intravenous TXA in orthopedic procedures are the reduction of intraoperative bleeding, transfusion volume, postoperative drain output volume, the score of pain perceived by the patient, and surgery complications [12–14]. The benefits mentioned are well systematically documented in the total knee [15, 16], hip [17], and shoulder arthroplasty [5, 18, 19]. Due to the scarcity of evidence to evaluate using TXA in elbow arthrolysis and a few included cases, the efficacy of TXA on elbow arthrolysis remains unclear. Furthermore, up to now, no systematic review or meta-analysis has not published on this topic. Therefore, in this systematic review and meta-analysis, we summarize and analyze the available studies on use of TXA in elbow arthrolysis, to assess the effects of TXA on drain output or postoperative blood loss, patients’ pain perception according to VAS pain score, postoperative elbow ROM, and postoperative complications.

## Methods

This systematic review and meta-analysis was performed according to Preferred Reporting Items for Systematic Reviews and Meta-Analyses (PRISMA) guidelines [20], and was registered in the PROSPERO prospective register of systematic reviews (ID: CRD42023447765).

### Search strategy and information source

On March 31^st,^ 2023 AHK searched the following phrases and keywords in PubMed, Web of Science, SCOPUS, and Cochrane library: ((((Tranexamic acid) OR (TXA)) OR (Transamine)) OR (TA)) AND (((Elbow arthroplasty) OR (Elbow arthrolysis)) OR (Elbow release)). We narrowed our search using English language and article document type. No additional filters were used.

### Eligibility criteria

The PICO form in our study was established as follows: Study population (P): open or closed elbow arthrolysis patients, intervention (I):intravenous TXA, comparison (C): placebo or no treatment, and outcomes (O): drain output, VAS pain score on day 1 after surgery, post-operative elbow ROM at the latest follow-up, and post-operative complications (hematoma, ulnar nerve palsy). We included controlled trials, either randomized (RCT) or non-randomized studies of intervention (NRSI) comparing the effects of intravenous tranexamic acid (TXA) treatment with placebo/no treatment on blood loss/other outcomes in patients that underwent elbow arthrolysis surgery (open/arthroscopic). Topical TXA, animal studies, cadaveric studies, non-comparative single group trials, review articles, non-English abstracts, letters, irrelevant, and articles with unavailable full text were excluded.

### Selection process and data extraction

Two reviewers (AHK and MHN) reviewed the search results, and screened based on title and abstract. Then proceeding to the full text screen of the remaining articles, we evaluated full-text regarding sufficient data, and none of them were excluded. Two of our team members (MHN and MD), extracted data and conducted the quality assessment. Data were extracted on the lead author, journal, year of publication, study design, study population, mean age, gender, surgical approach, administration mode and dosage, drain output, total blood loss, visual analog scale (VAS) pain score, range of motion (ROM), and complications (Tables 1 and 2).

**Table 1:** Included articles’ characteristics:

Author (year)	Study design	Group	Sample size	Surgery approach	Administration and dosage	Age (mean ± SD)	Gender M/F
Ju Tang, et al. (2018)	NRSI	Control	296	Open internal combined with external arthrolysis	Treated as in the case group except for treatment of TA	65.30 ± 4.11	133/163
		Intervention	291		15 mg/kg, 15 min before loose tourniquet + 500 mg infusion by drainage tube after suture	65.15 ± 3.52	125/166
Nitin Goyal, *et al. (2020)*	NRSI	Control	25	Open anterior and posterior joint releases	Treated as in the case group except for treatment of TA	45 ± 15	18/25
		Intervention	25		1 g within 30 min of incision + a single topical dose of TA (1 g in 20 mL saline solution) was infused through a deep hemovac drain after fascial closure.	45 ± 13	20/25
Eugene T. Ek, *et al. (2022)*	NRSI	Control	43	Arthroscopic osteocapsular release	Treated as in the case group except for treatment of TA	49.9 ± 13.3	33/10
		Intervention	40		1 g, completion of the arthroscopic procedure while the wounds were being closed, inflated tourniquet	45.9 ± 15.1	29/11
Haomin Cui, *et al. (2021)*	RCT	Control	48	Open elbow arthrolysis	100 mL saline, 10 min before skin incision	40 ± 10	35/13
		Intervention	48		1 g TXA in 100 mL saline, 10 min before skin incision	40 ± 12	28/20

NRSI: Non-randomized study of intervention, RCT: Randomized control trial study

**Table 2:** Included articles’ outcomes:

Author (year)	Study design	Groups	Drain output (ml)	Intraoperative blood loss (ml)	VAS pain score, day 1	ROM (degree)	Hematoma (N)	Ulnar nerve paralysis (N)
Ju Tang, et al. (2018)	NRCT	Control	450 ± 50	620 ± 50	---	---	18	16
		Intervention	420 ± 50	570 ± 50	---	---	7	12
Nitin Goyal, *et al. (2020)*	NRCT	Control	211 ± 134	37 ± 28	---	---		
		Intervention	121 ± 88	31 ± 21	---	---		
Eugene T. Ek, *et al. (2022)*	NRCT	Control	88.8 ± 80.5	--	1.9 ± 2.2	129.7 ± 12.4		
		Intervention	43.4 ± 52.4	--	1.5 ± 1.7	131.7 ± 9.2		
Haomin Cui, *et al. (2021)*	RCT	Control	214 ± 56	--	6 ± 1	120 ± 9	1	2
		Intervention	182 ± 46	--	5 ± 1	120 ± 7	1	3

NRCT: Non-randomized control trial study, RCT: Randomized control trial study

### Risk of biases assessment

We assessed the risk of biases in the studies using the revised Cochrane “Risk of bias” tool for randomized trials (RoB 2.0). RoB 2.0 addresses five specific domains: bias arising from (1) the randomization process, (2) interventions, (3) missing data outcome, (4) measurement of outcome, and (5) selection of the reported result. Each domain is assessed as low risk, some concern, or high risk. Then an overall judgment of the risk of biases is provided for each study. The risk of bias within NRSI studies was assessed using the Cochrane Risk of Bias in Non-randomized Studies – of Interventions (ROBINS-I) tool, a validated tool for assessing the quality of non-randomized studies. This tool assesses the risk of bias for confounding, participant selection, classification of interventions, deviations from intended interventions, missing data, outcome measurements, and selective reporting. Risk is quantified in each domain as low risk, moderate risk, serious risk, or critical risk, then an overall judgment of the risk of bias is provided for each study.

### Synthesis methods

The main outcome in our meta-analysis was an examination of the clinical outcome indicators following elbow arthrolysis with and without Tranexamic acid application, included postoperative blood loss, pain VAS score, elbow ROM, and complications. Forest plots were depicted to assess for heterogeneity and calculate the pooled weighted mean difference with corresponding 95% confidence intervals (WMD with 95% CI was used as pooled estimation of efficacy outcomes) for visual inspection across studies. Due to conceptual heterogeneity, a random-effects meta-analysis was conducted to account for the heterogeneity of the study populations. Pooled estimates with their corresponding 95% CIs were calculated using inverse-variance weights methods [21]. The I^2^ statistics was used to assess the heterogeneity across studies [22] (I^2^ = 0% indicates no observed heterogeneity and I2 ≥ 50% indicates substantial heterogeneity). Cochran’s Q statistic was also used to analyze the statistical significance of heterogeneity [23]. Sensitivity analysis was performed to determine which study (if any) had the largest impact on the heterogeneity and to assess the robustness of pooled estimates. Subgroup analyses, based on open or closed arthrolysis, were conducted. Visual inspection of funnel plots was done to assess publication bias (Fig. S1) [24]; WMD was plotted against the inverse of the square of the standard error. All statistical tests were two-tailed, and the significance level was set at less than 0.05 for all, except for the heterogeneity test. Statistical analyses were performed using Stata version 17.0 (Stata Corp., College Station, Texas, USA).

## Results

### Study selection

**Fig. 1:**
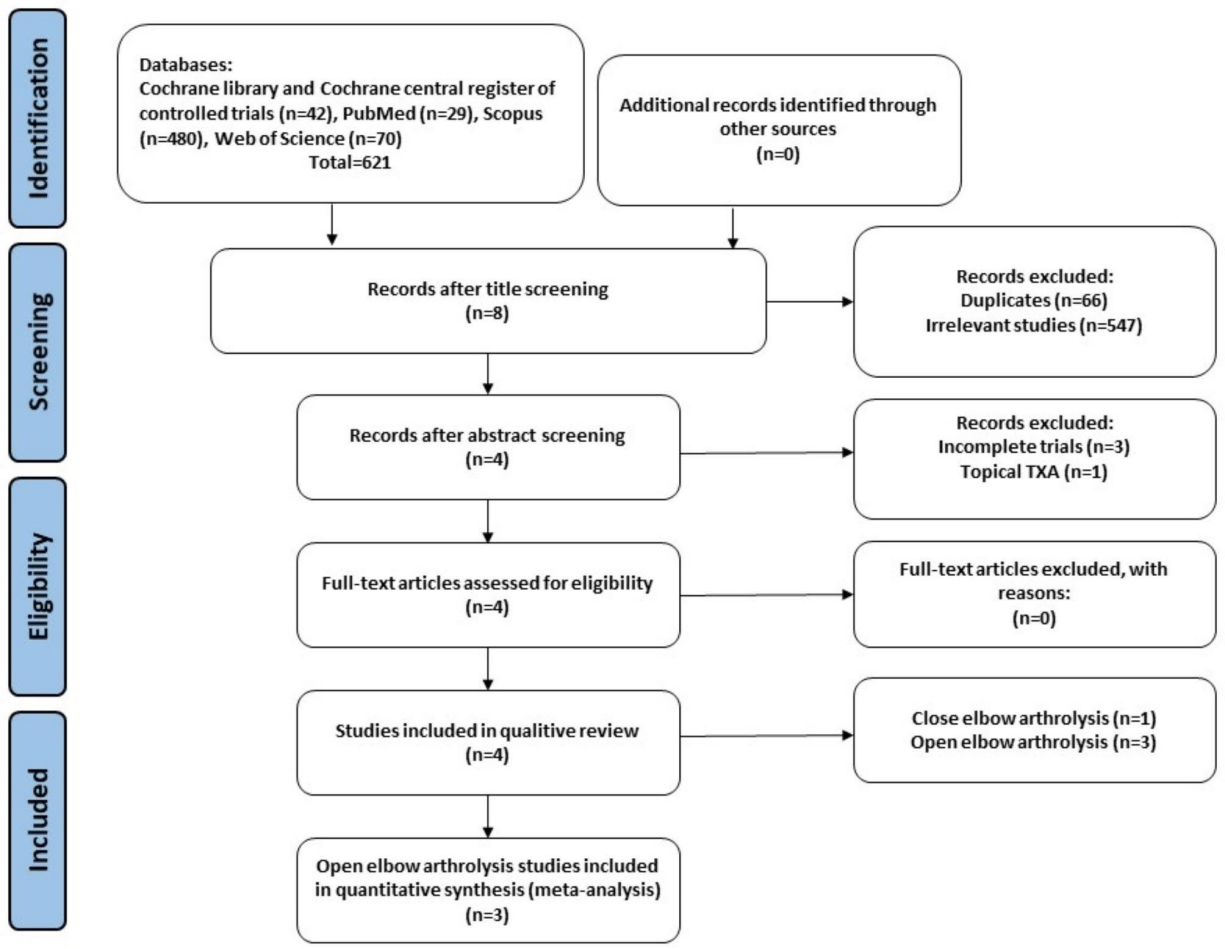
PRISMA flowchart

Our search strategy identified 621 potentially relevant citations, and this was reduced to 555 after duplicates were removed. After the initial screening of titles and abstracts, 4 full-text articles remained for further evaluation, and none failed to meet eligibility criteria. Therefore, four studies were deemed eligible for inclusion in this systematic review and meta-analysis on postoperative drain output (blood loss). About other variables of intraoperative blood loss, pain score and ROM, there were not sufficient homogeneous data to be analyzed. All four studies were related to elbow arthrolysis surgery [5–7, 25]. No studies on elbow arthroplasty were found. A PRISMA flow chart is provided in Fig. 1.

**Fig. 2:**
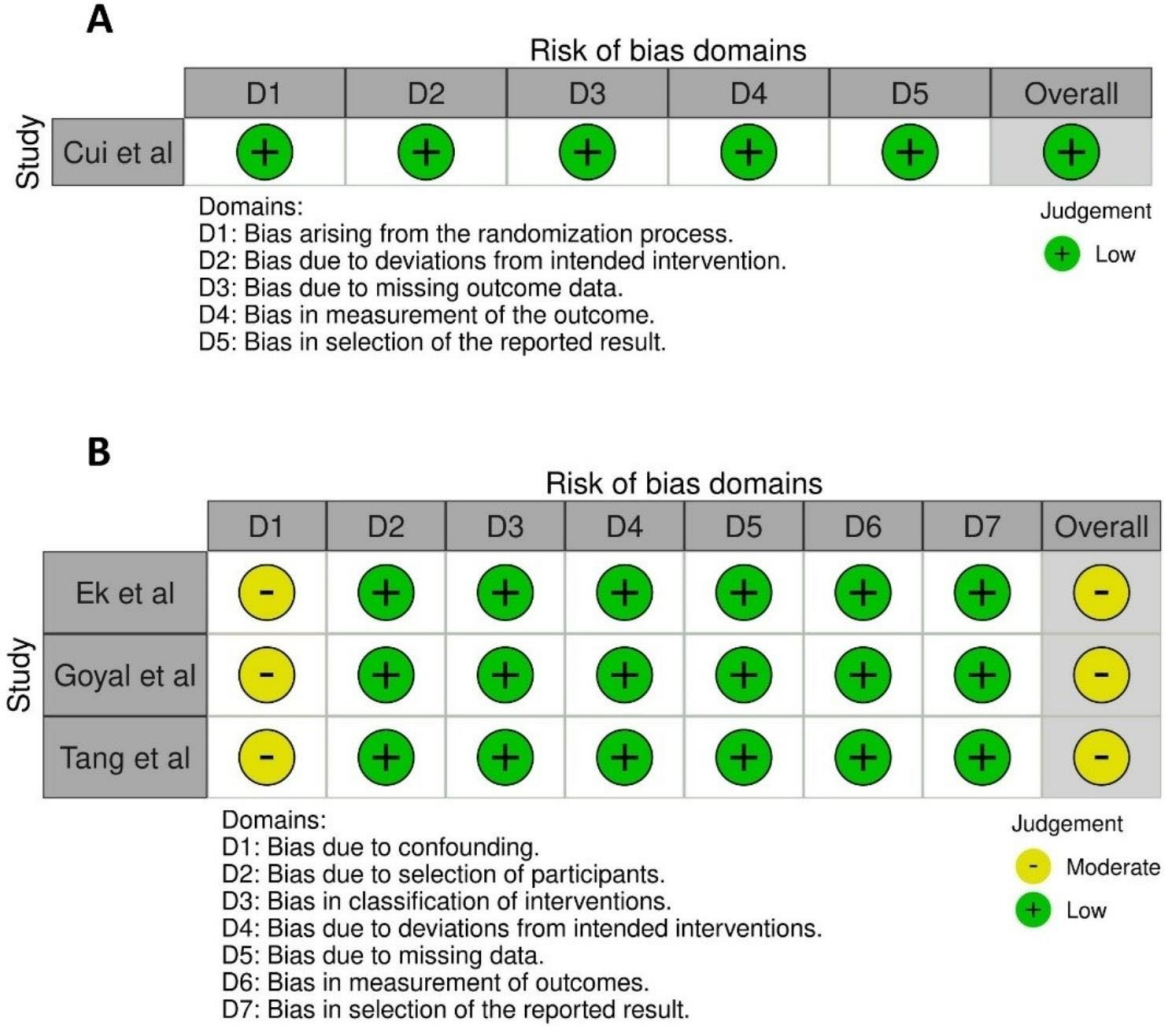
Risk of bias assessments **(A)** RoB 2 assessment of the RCT, **(B)** ROBINS-I assessment of NRCTs.

According to the RoB 2 risk of bias tool, the RCT was deemed to be of low risk of bias. The ROBINS-I tool indicated that NRSIs were assessed to be of moderate risk of bias. Analysis charts were produced by the Risk of Bias visualization tool (ROBVIS) [26] (Fig. 2).

### Study characteristics

Table 1 provides a summary of the characteristics of the included studies. One of the included studies was a RCT, and three were NRSI (all of them were conducted in China). All studies compared TXA (intravenous) to either no treatment or placebo (normal saline). All studies assessed post-operative blood loss in patients undergoing open or arthroscopic elbow arthrolysis.

The open surgical procedure involves multiple steps, including hardware removal, releasing anterior and posterior capsules, cleaning compartments, freeing ligaments, removing loose bodies, and potentially excising tips. Preferred approaches are medial and/or lateral, with common complications like temporary ulnar nerve dysfunction, reoperations for stiffness, and postoperative infections. In the arthroscopic technique, steps include debridement of compartments, eliminating loose bodies, excising the coronoid tip, and releasing the capsule. Persistent motion restriction is a significant complication, often requiring additional surgery [27].

All the studies included certain parameters for evaluation: drain output (mL) was reported in all studies, intraoperative blood loss was recorded in two studies, VAS pain score was documented in two studies, postoperative ROM in degrees was provided by two studies, and postoperative complications were indicated in one study. Specifically, Cui et al. compared TXA (1 g administered 10 min before skin incision) (n = 48) against placebo (100 mL saline administered 10 min before skin incision) (n = 48). Tang et al. compared TXA (15 mg/kg administered 15 min before applying a loose tourniquet + 500 mg infusion through a drainage tube after suturing) (n = 291) with no treatment (following the same procedure as the intervention group except for TXA) (n = 296). Goyal et al. compared TXA (1 g administered within 30 min of incision + 1 g topical dose of TXA in 20 mL saline solution infused through a deep hemovac drain after fascial closure) (n = 25) against no treatment (following the same procedure as the intervention group except for TXA) (n = 25). Lastly, Ek et al. compared TXA (1 g administered upon completion of the procedure while wounds were being closed with an inflated tourniquet) (n = 40) with no treatment (following the same procedure as the intervention group except for TXA) (n = 43).

#### Individual study results

As shown in Table 2, the results of each outcome measurement are as follows.

### Drain output (post-operative blood loss)

Drain output was used as a surrogate marker for post-operative blood loss, and was reported in all studies. In the TXA group, drain output was less than in the placebo group according to Cui, et al. (182 ± 46 mL vs.214 ± 56 mL; MD 32 mL, 95% CI 11, 53 mL; P = 0.003), Tang, et al. (420 ± 50 mL vs. 450 ± 50mL; P < 0.05), Goyal, et al. (121 ± 88 mL, range 15–360 mL vs. 221 ± 134 mL, range 50–580 mL; P = 0.003), and Ek, et al. data (43.4 ± 52.4 mL, range 0-190 mL vs. 88.8 ± 80.5 mL, range, 0-350mL; P = 0.0016) results.

### Intraoperative blood loss

In two studies, intraoperative blood loss was documented. Although Goyal et al. study indicated similar intraoperative blood loss between the TXA group and no-TXA group (31 ± 21 mL, range 10–100 mL vs. 37 ± 28 mL, range 5–100 mL; P = 0.476), Tang et al. study with much larger sample size, showed that intraoperative blood loss in the TXA group was significantly lower than that in the control group (570 ± 50 mL vs. 620 ± 50 mL; P < 0.05).

### VAS Pain score

The VAS score for pain was evaluated in two studies. Cui et al. data revealed that postoperative elbow pain was significantly less in the TXA group than in the placebo group on day 1 after surgery (5 ± 1 vs. 6 ± 1; MD 1, 95%CI 0, 1; P = 0.003). Despite, Ek et al. article shows no difference in pain level on day 1 between the no-TXA and TXA groups (1.9 ± 2.2, range 0–7 vs. 1.5 ± 1.7, range 0–4; P = 0.89).

### Range of motion (ROM)

Range of motion was reported in two studies [5, 28]. There was no significant difference between the no-TXA and TXA groups according to Cui et al. [5]. (120 °±9 ° vs. 120 ° ±7 °; MD 0, 95% CI 3, 4; P = 0.799) at six months’ follow-up, and Ek *et al. (28).* data (129.7 ± 12.4, range 80-145vs. 131.7 ± 9.2, range100-140; P = 0.549) at two months follow-up.

### Complications

Two of the reported complications were hematomas and ulnar nerve paralysis. In two studies, hematoma was documented. Cui et al. study revealed that both groups experienced similar incidences of subcutaneous hematoma after drain removal, but Tang et al. data, with larger sample size, revealed that the incidence of hematoma was higher in the control group compared to the TXA group (7 vs.18; P = 0.028). Ulnar nerve paralysis was also reported in two studies. Cui, et al. data showed no significant difference between participants of non-TXA and TXA groups regarding the development of ulnar nerve symptoms (2 vs. 3; P > 0.999) but Tang, et al. revealed more incidence of ulnar nerve paralysis in control group after surgery, but not significantly (12 vs.16; P = 0.466).

### Quantitative results (meta-analysis)

#### Postoperative drain output (ml)

**Fig. 3:**
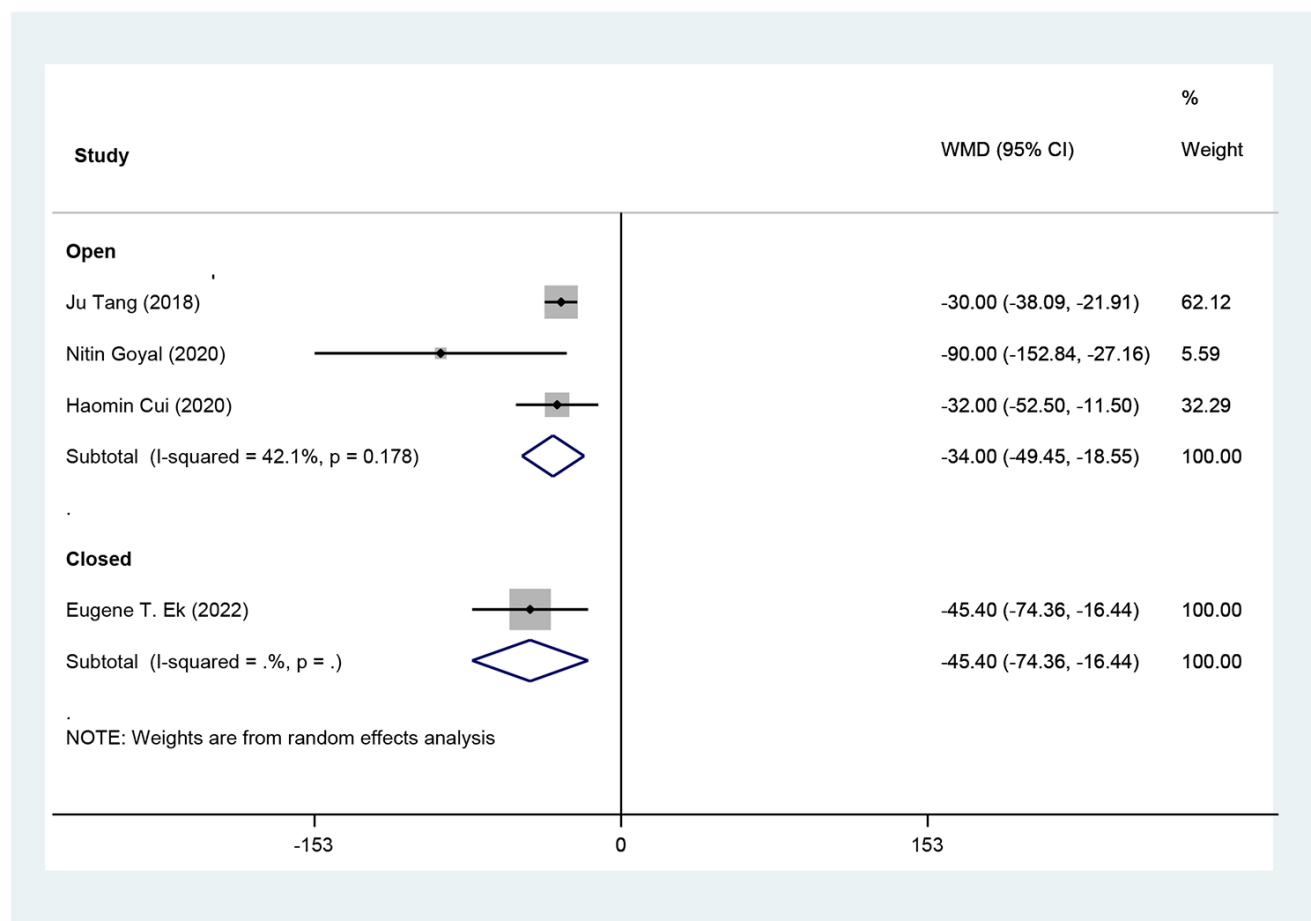
Forest plot of **drain output (ml)** for TXA group versus control group in patients who underwent elbow arthrolysis. Diamond represents the summary weighted mean difference (pooled WMD) estimate and its width shows corresponding 95% CI with random effects estimate. I2 test and Cochran’s Q statistic were used to assessing the statistical heterogeneity (P < 0.10) across studies

Analyzing three comparative studies with open elbow arthrolysis, comparing TXA group with control in terms of drain output, the weighted mean difference is -34.00 (95% CI: -49.45, -18.55), which means TXA application reduced drain output 34 mm on average (Fig. 3). Sensitivity analysis showed the mean change of drain output was consistent (range of summary WMDs: -43 to -31), indicating that the meta-analysis model was robust. The heterogeneity index (I^2^) was 42.1% which is not statistically significant (P = 0.178).

### Pooled estimation analysis

**Fig. 4:**
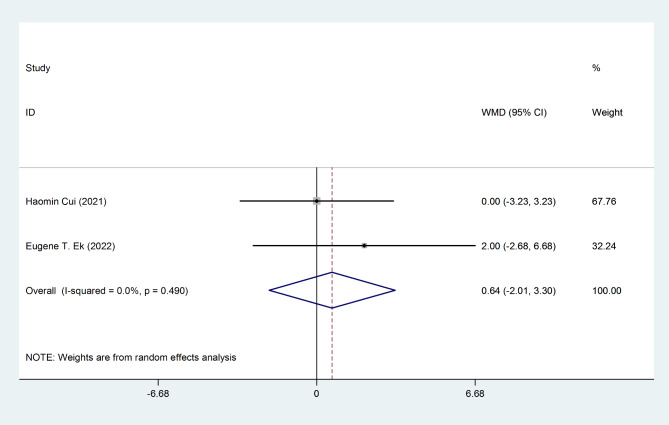
Forest plot of **ROM (degree)** for TXA group versus control group in patients who underwent elbow arthrolysis. Diamond represents the summary weighted mean difference (pooled WMD) estimate and its width shows corresponding 95% CI with random effects estimate

**Fig. 5:**
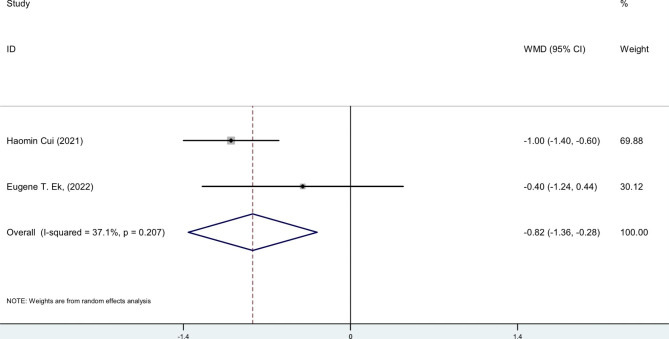
Forest plot of **VAS score** for TXA group versus control group in patients who underwent elbow arthrolysis. Diamond represents the summary weighted mean difference (pooled WMD) estimate and its width shows corresponding 95% CI with random effects estimate

**Fig. 6:**
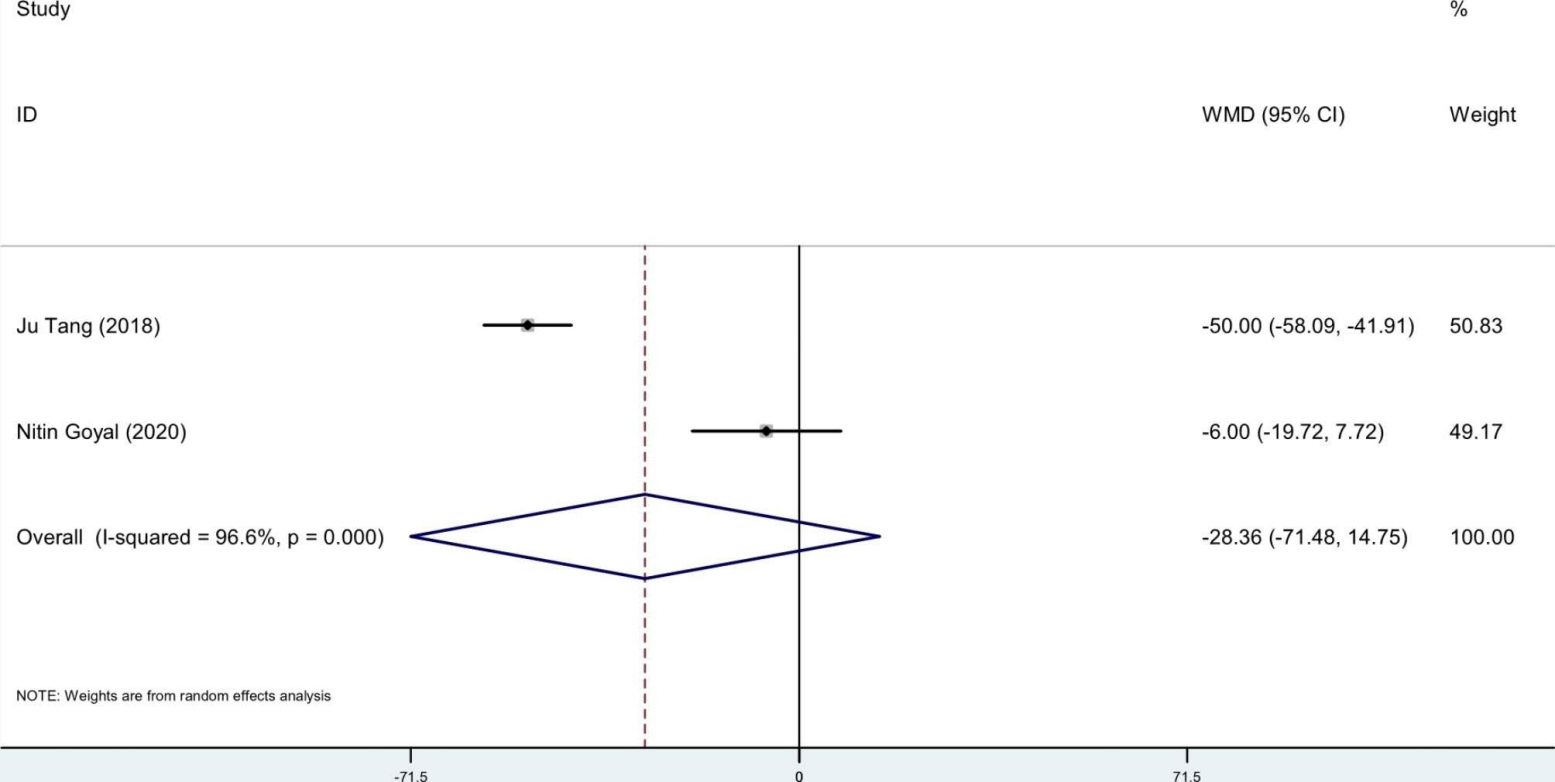
Forest plot of **intra-operative blood loss (ml)** for TXA group versus control group in patients who underwent elbow arthrolysis. Diamond represents the summary weighted mean difference (pooled WMD) estimate and its width shows corresponding 95% CI with random effects estimate

**Fig. 7:**
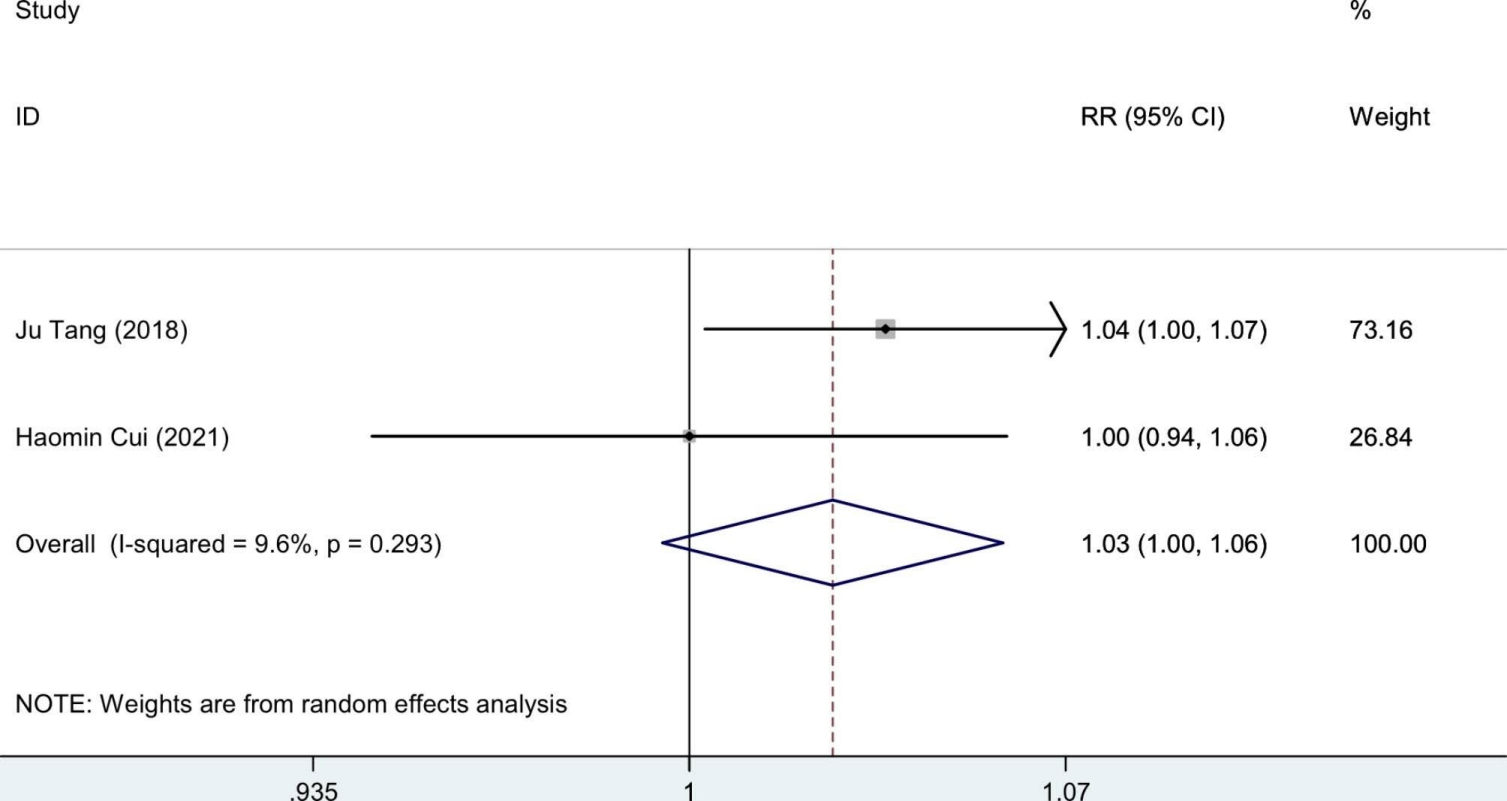
Forest plot of **hematoma rate** for TXA group versus control group in patients who underwent elbow arthrolysis. Diamond represents the summary risk ratio (pooled RR) estimate and its width shows corresponding 95% CI with random effects estimate

**Fig. 8:**
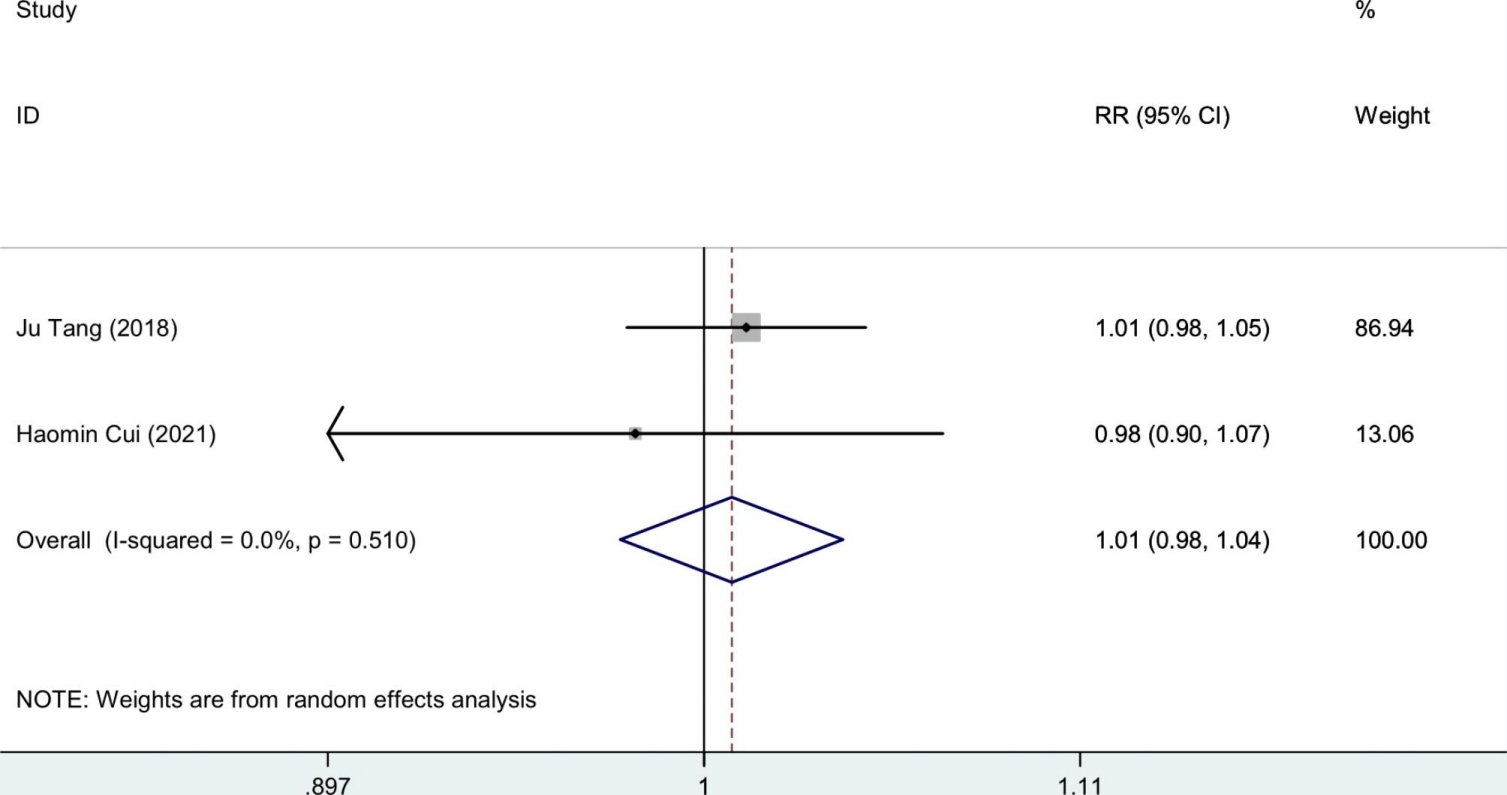
Forest plot of **ulnar nerve palsy rate** for TXA group versus control group in patients who underwent elbow arthrolysis. Diamond represents the summary risk ratio (pooled RR) estimate and its width shows corresponding 95% CI with random effects estimate

The pooled estimation of the two studies, comparing TXA group with control in terms of ROM, the weighted mean difference is 0.64 degrees (95% CI: -2.0, 3.3), which is not significant (Fig. 4). The pooled estimation of the two studies, comparing TXA group with control in terms of pain VAS score on day 1 post-operatively, the weighted mean difference is -0.82 score (95% CI: -1.36, -0.28), which is significant (Fig. 5). The pooled estimation of the two studies, comparing TXA group with control in terms of intra-operative blood loss, the weighted mean difference is -28.36 (95% CI: -71.48, 14.75), which is not significant (Fig. 6). The pooled estimation of the two studies, comparing TXA group with control in terms of intra-operative blood loss, the weighted mean difference is -28.36 (95% CI: -71.48, 14.75), which is not significant (Fig. 6). The complications’ rate of hematoma nad ulnar nerve palsy were not different between the two groups (Figs. 7 and 8).

## Discussion

Open and arthroscopic surgery are the two available procedures for elbow arthrolysis. The functional results of both open and arthroscopic elbow arthrolysis are acceptable; however, revisions and complications after open procedure rise dramatically. 6.3% of patients who underwent open arthrolysis required revision, while 18.1% experienced complications that did not necessitate revision surgery. On the other hand, individuals who underwent arthroscopic treatment had a revision rate of 1.6% and complications without revision of 9.1% [27, 29].

The present systematic review and meta-analysis main finding was that TXA significantly reduced drain output. This finding is in line with previous systematic reviews of other orthopedic procedures on hip [17, 30], shoulder [18, 31], and total knee arthroplasty [15, 16]. For instance, Kuo et al. investigated the effect of TXA compared to placebo on postoperative blood loss in shoulder arthroplasty and found a decrease of 95.41 ml. in drain output after 48 h in patients treated with TXA [31]. Our study is consistent with this finding, as we observed a reduction of 70% in postoperative blood loss.

The pain VAS score on the first postoperative day demonstrates a pain reduction among patients in the TXA group (Fig. 5). However, TXA did not yield a significant impact on elbow ROM (Fig. 4), intraoperative blood loss (Fig. 6), or postoperative complications (Figs. 7 and 8). The literature contains conflicting reports on the impact of TXA on postoperative range of motion (ROM) and improvements in pain as measured by the Visual Analog Scale (VAS) [32–36]. The proposed mechanism for faster recovery and pain relief suggests that bleeding during surgery causes inflammatory cells to migrate into the synovium, leading to inflammation and fibrosis at the surgical site. This can make the synovium more vulnerable to microtraumas and result in a cycle of bleeding and cartilage degeneration due to synovitis and arthrofibrosis. Additionally, the literature has documented direct cytotoxic effects of blood on cartilage matrix turnover [37]. Therefore, efficient hemostasis subsides inflammation, accelerates rehabilitation and reduces pain [5–7]. *Fried et al.*, designed and run a double-blind RCT to compare the efficacy of IV TXA in patients undergoing primary bone-patellar tendon-bone anterior cruciate ligament reconstruction and compared groups based on postoperative hemarthrosis, VAS pain score, opioid consumption, and ROM. Ultimately, they found no significant differences between groups [36]. Similarly, *Nugent et al.*, enrolled patients undergoing arthroscopic meniscectomy in a double-blind RCT, and compared ROM, and VAS pain scores between the control and TXA group. They observed no significant difference between the control and TXA groups in the mentioned variables at 3, 14, and 30 days of follow-up [38]. Conversely, Mackenzie and colleagues found no notable difference between the groups in terms of the VAS pain score three days after rotator cuff repair surgery. However, the TXA group showed significantly improved VAS pain scores after eight weeks and significantly greater range of motion (ROM) after six months [35]. *Peters et al.*, also showed significantly lower VAS pain score and higher ROM at the time of discharge in TXA group in open wedge high tibial osteotomy surgery [39]. The plasmin profiles of different body parts in pigs vary, and this may be true for humans as well. Our hypothesis is that several factors contribute to the conflicting findings, such as the specific procedures performed, technical considerations, length of the follow-up period, data collection methods, and the anatomical sites involved [10].

Surprisingly, there is no documented optimal dose, route, timing, or formulation for TXA administration. In our study, these variables vary, and impact the drain output, differently. Fillingham et al. conducted a network meta-analysis to assess the effectiveness of TXA in reducing blood loss in total knee arthroplasty, considering various doses, formulations, and timing of administration. They discovered that TXA, regardless of its formulation, timing, or number of doses, with the exception of oral TXA, significantly reduces blood loss. However, they were unable to determine any clear advantages in terms of dosage, timing, or formulation for blood loss reduction. Nevertheless, they suggest that pre-incisional IV TXA injection is a preferable option based on the moderate level of evidence [16].

Similarly, Sershon et al. conducted a study on the optimal dose of the tranexamic acid in revision total hip arthroplasty. They examined four different dosing and timing methods, which included giving a single dose of 1 g IV tranexamic acid before incision, administering 1 g IV tranexamic acid before incision followed by another 1 g IV dose after arthrotomy wound closure, using a combination of 1 g IV tranexamic acid before incision and 1 g topical tranexamic acid during surgery, or administering three oral doses totaling 1,950 mg of tranexamic acid. Ultimately, the researchers concluded that there was no significant variation in the effectiveness of the four methods in terms of reducing levels of Hb, blood loss, and transfusion requirements [30]. Based on the included studies, the advised and frequently utilized dosage of TXA for elbow arthrolysis, either open or arthroscopic, stands at 1gr (equivalent to 10 to 15 mg/kg), to be administered before the initiation of skin incision and/or during the process of skin closure [5, 28, 40, 41]. However, certain studies propose a more effective method of prescribing. *Ravi Saravanan et al.*, conducted a comparison of the effectiveness of various dosages and timing of TXA in major orthopedics surgeries. Patients were divided into five groups: low dose (bolus 10 mg/kg), low dose + maintenance (bolus 10 mg/kg + maintenance 1 mg/kg/hr), high dose (bolus30 mg/kg) and high dose + maintenance (bolus 30 mg/kg + maintenance 3 mg/kg/hr). The Bolus dose was injected prior to the incision, and infusion and maintenance continued until the end of the surgery. Ultimately, they concluded that perioperative blood loss and transfusion requirements were significantly reduced when the maintenance infusion was used in conjunction with a bolus dose regardless of a higher or lower dose [42]. *Balachandar et al.*, compared the preoperative and postoperative effectiveness of IV TXA administration in bilateral total knee arthroplasty surgery. The findings indicated that receiving an IV injection of TXA during surgery resulted in a notably lower reduction in hemoglobin levels on the first day following the operation. However, there was no substantial impact on the need for transfusions or the amount of fluid drainage [43]. It is assumed that TXA operates under the all-or-nothing rule, which means that the drug is ineffective if its serum concentration falls below a certain threshold. This is why intravenous infusion is more effective than multiple dosages, as it keeps the serum level above the threshold throughout the surgery. According to this principle, increasing the dose will not improve performance since even low doses of tranexamic acid can produce effects. The oral form of the drug is less potent than the intravenous form because it reaches the therapeutic levels within an hour, whereas the intravenous form reaches only after a few minutes. Thus, the oral form is somewhere in the middle of the potency spectrum between the intravenous form and a placebo [44].

This study is limited by several factors. First, the few number of the homogenous studies, and that three of them were not randomized and further prospective RCT studies with larger sample sizes and longer follow up duration are needed. Second, the search was limited to available English studies. Third, the included studies differed in postsurgical timing and the accuracy of measuring drain output. Furthermore, studies were heterogenic regarding administration dose and frequency of TXA. Calculating sample size based on postoperative drainage, reduces the power of the study to detect significance in secondary outcomes such as postoperative complications.

## Conclusion

The present systematic-review and meta-analysis on the efficacy of TXA in blood loss during elbow arthrolysis surgery has demonstrated that perioperative administration of TXA reduces post-operative bleeding. Due to the lack of sufficient high-quality RCT articles, further studies are needed for a robust conclusion.

### Electronic supplementary material

Below is the link to the electronic supplementary material.

Supplementary Material 1

Supplementary Material 2

Supplementary Material 3

Supplementary Material 4

Supplementary Material 5

Supplementary Material 6

Supplementary Material 7

Supplementary Material 8

Supplementary Material 9

Supplementary Material 10

## Data Availability

The data that support the findings of this study are available from Ali Moradi: almor0012@gmail.com.

## Declarations

### Statement of the location

Present study was performed in Orthopedics Research Center, Ghaem hospital, Mashhad University of Medical Sciences (MUMS).

### Level of evidence

level I (systematic review and meta analysis).
