# Supplementary material for: Does tranexamic acid diminish hemorrhage and pain in open elbow arthrolysis? a systematic review and meta-analysis
Source: BMC Musculoskelet Disord. 2023 Oct 6;24:795. doi: 10.1186/s12891-023-06835-7 (PMC10557324; doi:10.1186/s12891-023-06835-7)
Supplement: Supplementary file 2 — Supplementary Material 2 [file 12891_2023_6835_MOESM2_ESM.docx]

**Figure S1**: Begg’s funnel plot for assessing the presence of publication bias. Weighted mean difference of drain output was plotted against the precision of the study (P=0.095, for Egger’s regression asymmetry test).
