# Supplementary material for: Does tranexamic acid diminish hemorrhage and pain in open elbow arthrolysis? a systematic review and meta-analysis
Source: BMC Musculoskelet Disord. 2023 Oct 6;24:795. doi: 10.1186/s12891-023-06835-7 (PMC10557324; doi:10.1186/s12891-023-06835-7)
Supplement: Supplementary file 7 — Supplementary Material 7 [file 12891_2023_6835_MOESM7_ESM.docx]

**Table S1:** Details of number of results from each syntax in Cochrane search engine

| **#** | **Search details** | **filters** | **Results** |
| --- | --- | --- | --- |
| 1 | tranexamic acid a | English | 3750 |
| 2 | txa | English | 1270 |
| 3 | transamine | English | 275 |
| 4 | ta | English | 14266 |
| 5 | elbow arthroplasty | English | 273 |
| 6 | elbow arthrolysis | English | 24 |
| 7 | elbow release | English | 352 |
| 8 | (tranexamic acid) OR (TXA) | English | 3802 |
| 9 | ((tranexamic acid) OR (TXA)) OR (transamine) | English | 4035 |
| 10 | (((tranexamic acid) OR (TXA)) OR (transamine)) OR (TA) | English | 17958 |
| 11 | ((((tranexamic acid) OR (TXA)) OR (transamine)) OR (TA)) AND (elbow arthroplasty) | English | 21 |
| 12 | ((((tranexamic acid) OR (TXA)) OR (transamine)) OR (TA)) AND ((elbow arthroplasty) OR (elbow arthrolysis)) | English | 27 |
| 13 | ((((Tranexamic acid) OR (TXA)) OR (Transamine)) OR (TA)) AND (((Elbow arthroplasty) OR (Elbow arthrolysis)) OR (Elbow release)) | English | 42 |
