# Supplementary material for: Does tranexamic acid diminish hemorrhage and pain in open elbow arthrolysis? a systematic review and meta-analysis
Source: BMC Musculoskelet Disord. 2023 Oct 6;24:795. doi: 10.1186/s12891-023-06835-7 (PMC10557324; doi:10.1186/s12891-023-06835-7)
Supplement: Supplementary file 8 — Supplementary Material 8 [file 12891_2023_6835_MOESM8_ESM.docx]

**Table S2:** Details of number of results from each syntax in Pubmed search engine

| **Search number** | **Query** | **Filters** | **Results** |
| --- | --- | --- | --- |
| 13 | ((((tranexamic acid) OR (TXA)) OR (transamine)) OR (TA)) AND (((Elbow arthroplasty) OR (elbow arthrolysis)) OR (Elbow release)) | English | 29 |
| 12 | ((((tranexamic acid) OR (TXA)) OR (transamine)) OR (TA)) AND ((Elbow arthroplasty) OR (elbow arthrolysis)) | English | 27 |
| 11 | ((((tranexamic acid) OR (TXA)) OR (transamine)) OR (TA)) AND (Elbow arthroplasty) | English | 26 |
| 10 | (((tranexamic acid) OR (TXA)) OR (transamine)) OR (TA) | English | 64,929 |
| 9 | ((tranexamic acid) OR (TXA)) OR (transamine) | English | 9,826 |
| 8 | (tranexamic acid) OR (TXA) | English | 7,695 |
| 7 | Elbow release | English | 1,681 |
| 6 | Elbow arthrolysis | English | 138 |
| 5 | Elbow arthroplasty | English | 4,969 |
| 4 | TA | English | 55,461 |
| 3 | Transamine | English | 2,143 |
| 2 | TXA | English | 3,098 |
| 1 | Tranexamic acid | English | 6,561 |
