# Supplementary material for: Does tranexamic acid diminish hemorrhage and pain in open elbow arthrolysis? a systematic review and meta-analysis
Source: BMC Musculoskelet Disord. 2023 Oct 6;24:795. doi: 10.1186/s12891-023-06835-7 (PMC10557324; doi:10.1186/s12891-023-06835-7)
Supplement: Supplementary file 9 — Supplementary Material 9 [file 12891_2023_6835_MOESM9_ESM.docx]

**Table S3:** Details of number of results from each syntax in Scopus search engine

| **Syntax** | **Results** |
| --- | --- |
| ( ( ( ( ALL ( tranexamic AND acid ) OR ALL ( txa ) ) OR ALL ( transamine ) ) OR ALL ( ta ) ) AND ( ( ALL ( elbow AND arthroplasty ) OR ALL ( elbow AND arthrolysis ) ) OR ALL ( elbow AND release ) ) ) AND ( LIMIT-TO ( DOCTYPE , "ar" ) ) AND ( LIMIT-TO ( LANGUAGE , "English" ) ) | 480 |
