# Supplementary material for: Does tranexamic acid diminish hemorrhage and pain in open elbow arthrolysis? a systematic review and meta-analysis
Source: BMC Musculoskelet Disord. 2023 Oct 6;24:795. doi: 10.1186/s12891-023-06835-7 (PMC10557324; doi:10.1186/s12891-023-06835-7)
Supplement: Supplementary file 10 — Supplementary Material 10 [file 12891_2023_6835_MOESM10_ESM.docx]

**Table S4:** Details of number of results from each syntax in Web of Science search engine

| **#** | **Search Query** | **Results** |
| --- | --- | --- |
| 1 | (ALL=(tranexamic acid)) AND LA=(English) | 5324 |
| 2 | (ALL=(TXA)) AND LA=(English) | 3803 |
| 3 | (ALL=(Transamine)) AND LA=(English) | 16 |
| 4 | (ALL=(TA)) AND LA=(English) | 344900 |
| 5 | (ALL=(elbow arthroplasty)) AND LA=(English) | 4100 |
| 6 | (ALL=(elbow arthrolysis)) AND LA=(English) | 155 |
| 7 | (ALL=(elbow release)) AND LA=(English) | 1622 |
| 8 | ((ALL=(tranexamic acid)) OR ALL=(TXA)) AND LA=(English) | 7615 |
| 9 | (((ALL=(tranexamic acid)) OR ALL=(TXA)) OR ALL=(transamine)) AND LA=(English) | 7627 |
| 10 | ((((ALL=(tranexamic acid)) OR ALL=(TXA)) OR ALL=(transamine)) OR ALL=(TA)) AND LA=(English) | 352139 |
| 11 | (((((ALL=(tranexamic acid)) OR ALL=(TXA)) OR ALL=(transamine)) OR ALL=(TA)) AND ALL=(elbow arthroplasty)) AND LA=(English) | 51 |
| 12 | (((((ALL=(tranexamic acid)) OR ALL=(TXA)) OR ALL=(transamine)) OR ALL=(TA)) AND ((ALL=(elbow arthroplasty)) OR ALL=(elbow arthrolysis))) AND LA=(English) | 55 |
| 13 | (((((ALL=(tranexamic acid)) OR ALL=(TXA)) OR ALL=(transamine)) OR ALL=(TA)) AND (((ALL=(elbow arthroplasty)) OR ALL=(elbow arthrolysis)) OR ALL=(elbow release))) AND LA=(English) | 70 |
